# The Elephant in the Operating Room: Disclosure of Payments From Industry

**DOI:** 10.1111/ans.70912

**Published:** 2026-08-10

**Authors:** Zakary Doherty, Travis Lines, Ian Harris, Malcolm Forbes

**Affiliations:** ^1^ Monash University Melbourne Victoria Australia; ^2^ Albury Wodonga Health Albury New South Wales Australia; ^3^ University of New South Wales Sydney New South Wales Australia; ^4^ Barwon Health Geelong Victoria Australia

Financial transparency is a cornerstone of maintaining public trust in the healthcare system, particularly regarding the relationships between doctors and industry. In Australia, the disclosure of industry‐to‐doctor payments serves as a critical lens through which these professional interactions can be understood. Using records from a database of pharmaceutical industry payments, we estimated the proportion of specialist doctors who received at least one payment from a pharmaceutical company over a nine‐year period, and report our results stratified by the medical college of which they are fellows (Figure 1) [1]. Between November 2015 and October 2024, 17.8% (16 337/91 698) of specialist doctors received ≥ 1 payment. There was wide variation between colleges, ranging from the Australasian College for Emergency Medicine (ACEM) with only 1.3% (48/3599) of fellows receiving a payment, through to the Australasian College of Dermatologists (ACD) with 52.8% (357/676) of fellows receiving a payment. The overall rate for fellows of the Royal Australasian College of Surgeons (RACS) was 6.9% (455/6572). When examining surgical subspecialties, wide variation was also seen; Urologists had the highest rate of payment (32.6% (170/521)) and Paediatric Surgeons had the lowest rate (0.9% (1/106)). The methods to obtain these figures are described in the (supplementary appendix in Supporting Information). These data provide an overview of financial interactions between doctors and the pharmaceutical industry. However, there is a notable exception: Payments from medical device companies are not included.

**FIGURE 1:**
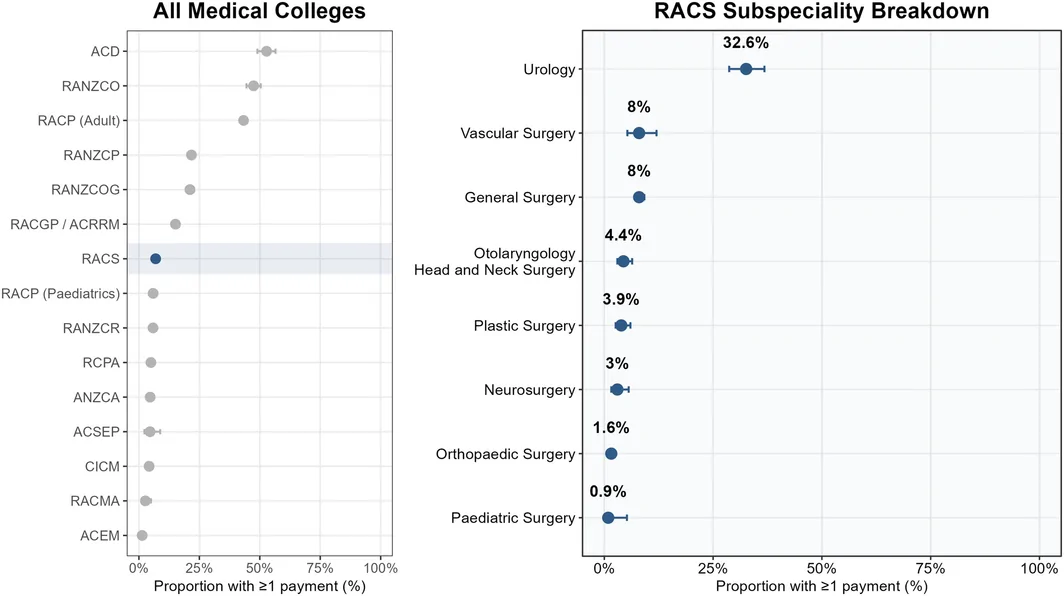
Pharmaceutical industry reach among Australian medical specialists, November 2015 to October 2024. Each point is the proportion of registered specialists in a given group who received at least one payment reported to Medicines Australia during the study period; horizontal bars are 95% Wilson confidence intervals. The left panel (‘All Medical Colleges’) shows reach by specialist college, with the Royal Australasian College of Surgeons (RACS) highlighted for comparison; the right panel (‘RACS Subspecialty Breakdown’) shows reach for the individual surgical specialties within RACS, ordered from highest to lowest. Denominators are end‐of‐period specialist workforce counts from the Australian Health Practitioner Regulation Agency 2023/24 annual report. Payments from medical device companies are not captured by the Medicines Australia scheme and are therefore excluded. College abbreviations are defined in (Table S1).

Payment reporting is managed by Medicines Australia, the peak pharmaceutical industry association in Australia. Reporting began in November 2015 and since 2019, payment reports have been collated in a publicly available database. The Australian Competition and Consumer Commission (ACCC) required these reports as a condition of their authorisation of the Medicines Australia Code of Conduct [2]. The equivalent body for medical device companies is the Medical Technology Association of Australia (MTAA). The MTAA has not sought ACCC authorisation for its Code of Practice, nor does it require payments from member companies to doctors to be publicly disclosed [3]. There is no legislation requiring mandatory disclosure of device industry payments in Australia. Consequently, the extent of financial relationships between Australian doctors and medical device companies remains unknown.

Internationally, this is not the case. In the United States (US), the Physician Payments Sunshine Act has ensured that all industry payments, including those from medical device companies, have been public since 2013. Concern regarding high payment rates and lack of a centralised reporting system were the principal triggers for the Act [4]. Following the introduction of the database it was found over 60% of US surgeons between 2013 and 2022 received industry payments, nearly 10 times higher than the reported rate for Australian surgeons, which does not include payments from medical device companies [5]. A notable example is orthopaedic surgery: In Australia, our data show that only 1.6% (26/1590) of orthopaedic surgeons received payments, whereas in the US, that figure is approximately 65%. Overall, in the US, a total of $2.7 billion (USD) was paid to doctors by medical device companies between 2013 and 2022 (personal communication, Professor Joseph Ross, Yale University). While the market is smaller in Australia, it can be safely assumed that tens of millions of dollars in payments from medical device companies to procedural specialists have been made over the past decade. However, how much and to whom is unknown, in turn preventing accurate estimates of overall payment rates from industry to Australian doctors.

Payments are made to influence behaviour [1]. Even small exchanges matter, with observational evidence indicating that a single sponsored meal is associated with increased prescribing of the promoted brand [6]. This association has also been identified for device implantation, specifically cardiac devices [7]. Perhaps more consequential is the association between financial conflicts of interest and recommendations in clinical guidelines, by virtue of their potential to impact the behaviour of the group accessing them [8]. A number of measures exist to mitigate possible influence resulting from payments, including guidance from the National Health and Medical Research Council on how to manage conflicts of interest in guideline development [9]. However, the effect of these measures is unknown. The specific strategy of mandatory payment disclosures has been associated with a decrease in both the total value and number of payments in the US [10]. While the causal impact of mandatory payment disclosures remains difficult to disentangle, there is a compelling case that information should be publicly available to aid decision‐making by patients. Currently, RACS recommends that surgeons declare industry relationships before recommending services [11]. However, the reasons for payments are often complex, covering travel for educational events, technical training for new devices or compensation for specific expertise. Relying solely on verbal disclosure during a time‐limited consultation places an undue burden on both the clinician to explain and the patient to interpret these interests in real‐time. A freely accessible and centralised online database containing information on all payments made from both the pharmaceutical and medical device industry is required. To preserve independence, we believe the Australian Government is best positioned to collect, maintain and publish these records, ideally established in legislation. Such a database would provide patients, healthcare professionals and organisations, regulators, researchers and governments with access to comprehensive payment information.

Australia currently operates a two‐tier transparency scheme: Payments from pharmaceutical companies are publicly disclosed, while medical device payments remain unreported. This gap is most significant for surgical and procedural specialties. Financial relationships with industry, whether related to pharmaceutical or medical device companies are not inherently positive or negative; however, inadequate knowledge of industry‐doctor relationships may influence clinical practice, limit informed consent and weaken conflict of interest management in guideline development. Reforming the disclosure scheme to include payments from both the pharmaceutical and medical device industry in an independent and centralised database would align Australia with contemporary best practice and provide a complete account of the professional relationships between doctors and industry [12].

## Author Contributions

**Zakary Doherty:** conceptualization, writing – original draft, methodology, visualization, writing – review and editing, formal analysis. **Malcolm Forbes:** writing – review and editing, writing – original draft, conceptualization, data curation, supervision. **Travis Lines:** conceptualization, writing – original draft, methodology, writing – review and editing. **Ian Harris:** supervision, writing – review and editing, writing – original draft.

## Supporting information


**Table S1:** List of specialties and colleges.

## Data Availability

The data that support the findings of this study are openly available in surgical_payments_dataset at https://github.com/zbdoh1/Pharma‐Payments‐Sugical.
