# Supplementary material for: The Elephant in the Operating Room: Disclosure of Payments From Industry
Source: ANZ J Surg. 2026 Aug 10;96(10):2738–40. doi: 10.1111/ans.70912 (PMC13724755; doi:10.1111/ans.70912)
Supplement: Supplementary file 1 [file ANS-96-2738-s001.docx]

**Supplementary Appendix**

**Supplementary Methods**

Data on payments to health professionals from November 2019 to October 2024 were downloaded from the centralised Medicines Australia repository.^1^ These reports include individual payment records with fields for company name, practitioner name, healthcare professional type, practice address, service type, event type, payment recipient, registration fees, travel costs, fees for service, and reporting period. These data were merged with legacy reports (October 2015–October 2019) obtained from the University of Sydney repository.^2^

To assign each doctor to a specialty, a manual search of the Ahpra Register of Practitioners was performed.^3^ When an exact match was unclear, for example, for common names shared by multiple clinicians, additional verification was undertaken using hospital or clinic websites, conference speaker biographies, and other publicly available sources. In some cases, practitioners held more than one specialty; to avoid double-counting, each individual was assigned a single primary specialty, determined through targeted online searches. Further details on the database construction are available elsewhere.^4^

Each clinician’s primary specialty was then mapped to the relevant specialist college using the list of approved programs of study from Ahpra. ^5^ For example, a practitioner registered as a Specialist in Anaesthesia was assigned to the Australian and New Zealand College of Anaesthetists. The full list of specialties and corresponding colleges is provided in Table S1. Some specialties require dual fellowship; for the purposes of this analysis the following allocations were applied:

1. Haematology - RACP
2. Microbiology - RACP
3. Immunology/Allergy - RACP

For each specialty we estimated pharmaceutical company reach (the proportion of registered clinicians in each specialty who received ≥ 1 payment) by linking payments to AHPRA 2023/24 workforce counts.^6^ The number of specialists in each speciality at the end of the study period was used to calculate proportions. This provides the most conservative estimate of pharmaceutical company reach. Proportions and 95% Wilson Confidence Intervals were calculated for Figure 1. In the first part of Figure 1 College level results were presented, next specialties under the Royal Australasian College of Surgeons (RACS) were analysed individually.

Formal data analysis was undertaken by M.F. and Z.D. using R version 4.4.2, with independent checking by T.L. Visualisation was completed by Z.D. All code and data are available at: [https://github.com/zbdoh1/Pharma-Payments-Surgical)](https://github.com/zbdoh1/Pharma-Payments-by-College). The released analysis dataset excludes practitioner address fields to protect privacy, as some individuals listed personal rather than professional addresses. This study uses publicly available data and the data utilised for this study was previously determined to be exempt from review by The University of Sydney Human Research Ethics Committee. This research did not receive any specific grant from funding agencies in the public, commercial, or not-for-profit sectors.

**Supplementary Table 1:**

| **College Acronym** | **College Name** | **Speciality** |
| --- | --- | --- |
| ACD | Australasian College of Dermatologists | Dermatology |
| ACEM | Australasian College of Emergency Medicine | Emergency Medicine |
| RANZCO | Royal Australian and New Zealand College of Ophthalmologists | Ophthalmology |
| RACP (Adult) | Royal Australasian College of Physicians (Adult Medicine Division) | Cardiology; Clinical Genetics; Clinical Pharmacology; Endocrinology; Gastroenterology & Hepatology; General Medicine; Geriatric Medicine; Haematology; Immunology & Allergy; Infectious Diseases; Medical Oncology; Nephrology; Neurology; Nuclear Medicine; Respiratory & Sleep Medicine; Rheumatology; Rehabilitation Medicine; Sexual Health Medicine; Public Health Medicine; Occupational & Environmental Medicine; Addiction Medicine; Palliative Medicine |
| RANZCP | Royal Australian and New Zealand College of Psychiatrists | Psychiatry |
| RANZCOG | Royal Australian and New Zealand College of Obstetricians & Gynaecologists | Obstetrics & Gynaecology (inc. Gyn Onc; Maternal–Fetal Medicine; Reproductive Endocrinology & Infertility; Ultrasound; Urogynaecology) |
| RACGP/ACRRM | Royal Australian College of General Practitioners / Australian College of Rural & Remote Medicine | General Practice |
| RACS | Royal Australasian College of Surgeons | Cardiothoracic Surgery; General Surgery; Neurosurgery; Orthopaedic Surgery; Otolaryngology - Head & Neck Surgery; Paediatric Surgery; Plastic Surgery; Urology; Vascular Surgery |
| RACP (Paediatric) | Royal Australasian College of Physicians (Paediatrics & Child Health Division) | General Paediatrics; Clinical Genetics; Community Child Health; Neonatal & Perinatal Medicine; Paediatric Cardiology; Paediatric Clinical Pharmacology; Paediatric Emergency Medicine; Paediatric Endocrinology; Paediatric Gastroenterology & Hepatology; Paediatric Haematology; Paediatric Immunology & Allergy; Paediatric Infectious Diseases; Paediatric Intensive Care; Paediatric Medical Oncology; Paediatric Nephrology; Paediatric Neurology; Paediatric Nuclear Medicine; Paediatric Palliative Medicine; Paediatric Rehabilitation Medicine; Paediatric Respiratory & Sleep Medicine; Paediatric Rheumatology |
| RANZCR | Royal Australian and New Zealand College of Radiologists | Diagnostic Radiology; Radiation Oncology; Nuclear Medicine (Radiology) |
| RCPA | Royal College of Pathologists of Australasia | Anatomical Pathology; Chemical Pathology; Forensic Pathology; General Pathology; Haematology; Immunology; Microbiology (Haematology, Immunology and Microbiology were allocated to RACP). |
| CICM | College of Intensive Care Medicine of Australia & New Zealand | Intensive Care Medicine |
| ANZCA | Australian and New Zealand College of Anaesthetists | Anaesthesia, Pain Medicine |

**Supplementary Table 1.** **List of specialties and colleges.**

**Supplementary References:**

1. Medicines Australia. Transparency reporting [Internet]. Canberra: Medicines Australia; 2022 [cited 2026 Jul 25]. Available from: <https://www.medicinesaustralia.com.au/search>
2. Bero L, Mintzes B, Grundy Q, Fabbri A, Williams J, Parker L. Pharmaceutical industry payments to healthcare professionals, May 2016–April 2017 [dataset on the Internet]. Sydney: University of Sydney; 2019 [cited 2026 Jul 25]. Available from:<https://ses.library.usyd.edu.au/handle/2123/20217>
3. Australian Health Practitioner Regulation Agency. Register of practitioners [Internet]. Melbourne: Ahpra; [cited 2026 Jul 25]. Available from:<https://www.ahpra.gov.au/Registration/Registers-of-Practitioners.aspx>
4. Forbes M, Harvey K, Schien S, Hooimeyer A, Pokorny A. Pharmaceutical payments to Australian health care professionals from 2015 to 2024. *Sci Rep.* 2026; in press. doi:10.1038/s41598-026-62851-2
5. Australian Health Practitioner Regulation Agency. Approved programs of study: medical practitioner—specialist [Internet]. Melbourne: Ahpra; 2026 [cited 2026 Jul 25]. Available from:<https://www.ahpra.gov.au/Accreditation/Approved-Programs-of-Study.aspx?ref=Medical%20Practitioner&type=Specialist>
6. Australian Health Practitioner Regulation Agency. Annual report 2023/24: Table R9—health practitioners with specialties [Internet]. Melbourne: Ahpra; 2024 [cited 2026 Jul 25]. Available from:<https://www.ahpra.gov.au/Publications/Annual-reports/Annual-report-2024/Index-of-tables.aspx>
